# Effects of ground and joint reaction force exercise on lumbar spine and femoral neck bone mineral density in postmenopausal women: a meta-analysis of randomized controlled trials

**DOI:** 10.1186/1471-2474-13-177

**Published:** 2012-09-20

**Authors:** George A Kelley, Kristi S Kelley, Wendy M Kohrt

**Affiliations:** 1 Meta-Analytic Research Group, School of Public Health, Department of Biostatistics, Robert C. Byrd Health Sciences Center, West Virginia University, Morgantown, 26506-9190, WV, USA; 2 Division of Geriatric Medicine, University of Colorado Denver, Anschutz Medical Campus, 12631 East 17th Avenue - L15, PO Box 6511, Mail Stop B179, Aurora, 80045, CO, USA

**Keywords:** Exercise, Bone, Osteoporosis, Women, Postmenopausal, Aging, Meta-analysis, Systematic review

## Abstract

**Background:**

Low bone mineral density (BMD) and subsequent fractures are a major public health problem in postmenopausal women. The purpose of this study was to use the aggregate data meta-analytic approach to examine the effects of ground (for example, walking) and/or joint reaction (for example, strength training) exercise on femoral neck (FN) and lumbar spine (LS) BMD in postmenopausal women.

**Methods:**

The *a priori* inclusion criteria were: (1) randomized controlled trials, (2) exercise intervention ≥ 24 weeks, (3) comparative control group, (4) postmenopausal women, (5) participants not regularly active, i.e., less than 150 minutes of moderate intensity (3.0 to 5.9 metabolic equivalents) weight bearing endurance activity per week, less than 75 minutes of vigorous intensity (> 6.0 metabolic equivalents) weight bearing endurance activity per week, resistance training < 2 times per week, (6) published and unpublished studies in any language since January 1, 1989, (7) BMD data available at the FN and/or LS. Studies were located by searching six electronic databases, cross-referencing, hand searching and expert review. Dual selection of studies and data abstraction were performed. Hedge’s standardized effect size (*g*) was calculated for each FN and LS BMD result and pooled using random-effects models. Z*-score* alpha values, 95%confidence intervals (CI) and number-needed-to-treat (NNT) were calculated for pooled results. Heterogeneity was examined using Q and *I*^*2*^. Mixed-effects ANOVA and simple meta-regression were used to examine changes in FN and LS BMD according to selected categorical and continuous variables. Statistical significance was set at an alpha value ≤0.05 and a trend at >0.05 to ≤ 0.10.

**Results:**

Small, statistically significant exercise minus control group improvements were found for both FN (28 *g’s*, 1632 participants, *g* = 0.288, 95% CI = 0.102, 0.474, *p* = 0.002, Q = 90.5, *p* < 0.0001, *I*^*2*^ = 70.1%, NNT = 6) and LS (28 *g’s*, 1504 participants, *g* = 0.179, 95% CI = −0.003, 0.361, *p* = 0.05, Q = 77.7, *p* < 0.0001, *I*^*2*^ = 65.3%, NNT = 6) BMD. Clinically, it was estimated that the overall changes in FN and LS would reduce the 20-year relative risk of osteoporotic fracture at any site by approximately 11% and 10%, respectively. None of the mixed-effects ANOVA analyses were statistically significant. Statistically significant, or a trend for statistically significant, associations were observed for changes in FN and LS BMD and 20 different predictors.

**Conclusions:**

The overall findings suggest that exercise may result in clinically relevant benefits to FN and LS BMD in postmenopausal women. Several of the observed associations appear worthy of further investigation in well-designed randomized controlled trials.

## Background

Osteoporosis is a major public health problem affecting an estimated 200 million women worldwide [1]. Congruent with osteoporosis is an increased risk for osteoporosis-related fractures, especially in women during the postmenopausal years, generally considered to begin around 50 years of age [2]. Comparatively, the lifetime risk of an osteoporosis-related fracture in women is equivalent to the risk of developing cardiovascular disease [3]. The two most common sites for osteoporosis-related fractures are the hip and the spine, with an estimated worldwide prevalence of 1.1 million and 862,000, respectively, in women 50 years of age and older in the year 2000 [2]. In the United States, the total annual costs associated with osteoporosis-related fractures were more than $19 billion in 2005 with a predicted increase to $25.3 billion in 2025 [4]. The majority of the costs in 2005 were attributed to fractures of the hip (72%) followed by the spine (6%) [4].

Prevention of osteoporosis has focused on maximizing bone mineral density (BMD) during childhood and adolescence and maintaining BMD during adulthood [5,6]. Preventive measures include adequate calcium and vitamin D intake as well as avoiding cigarette smoking and excessive alcohol intake [5,6]. In addition, ground reaction (for example, jogging) and joint reaction (for example, strength training) force exercise has been recommended across the lifespan [5-8]. However, the results of previous randomized controlled exercise intervention trials have reached conflicting and underwhelming conclusions regarding the effects of ground reaction and/or joint reaction force exercise on BMD at the femoral neck (FN) and lumbar spine (LS) in postmenopausal women [9-33]. For example, using the vote-counting approach, only 29% of the exercise versus control group differences in FN BMD have been reported as statistically significant and in the direction of benefit while even fewer (11%) have been reported at the LS [9-33]. Based on these findings, one might reach the general conclusion that ground and joint reaction force exercise have little or no effect on FN and LS BMD. However, reliance on a vote-counting approach based on statistical significance can be extremely misleading since the absence of a statistically significant effect does not mean that an effect is absent [34]. In contrast, meta-analysis allows one to go beyond statistical significance and focus on the magnitude of effect. It is a quantitative approach for combining the results of studies. The strengths of meta-analysis include: (1) increased power, (2) improved estimates of effect size (ES), and (3) the potential to resolve disagreements between studies [35].

While a number of meta-analyses have been conducted on the effects of exercise on FN and LS BMD in adults [36-54], fewer have focused, or partitioned data, according to randomized controlled trials in postmenopausal women [37,39,48-51,53]. One meta-analysis that included studies published up to December, 1995 found a statistically significant exercise minus control group benefit of 0.73% in LS BMD as a result of joint and/or ground reaction force exercise in postmenopausal women [39]. Another meta-analysis that included studies published up to January, 1998 reported a statistically significant benefit in FN and LS BMD ranging from 0.9% to 1.6% as a result of impact and non-impact exercise among postmenopausal women [53]. However, both meta-analyses were limited to studies published more than 14 years ago. Since that time, additional randomized controlled trials with inconsistent findings have been published [10-12,14-17,19-22,24-27,30,32,33]. In addition, guidelines for the improved conduct of meta-analysis have been developed [47].

A modality-specific, joint reaction force meta-analysis that included studies published up to December 2004 found a statistically significant benefit of 0.006 g/cm^2^ in LS BMD and a non-significant benefit of 0.010 g/cm^2^ in FN BMD as a result of high-intensity resistance exercise in postmenopausal women [49]. Another modality-specific meta-analysis by the same research group which included studies published through December 2006 reported a non-statistically significant benefit in FN and LS BMD in postmenopausal women as a result of walking [50]. These findings suggest that walking, a lower impact, ground reaction force exercise, may have little benefit on FN and LS BMD in postmenopausal women. The same research group also published another meta-analysis that included studies published to 2008 [51]. When limited to randomized controlled trials and a random-effects model, a statistically significant benefit of 0.004 g/cm^2^ was found for FN BMD with no statistically significant benefit observed at the LS as a result of exercise in postmenopausal women [51]. More recently, a Cochrane systematic review by Howe et al. reported a statistically significant exercise minus control group benefit of 0.85% in LS BMD but no significant change in FN BMD (−0.08%) as a result of joint and/or ground reaction force exercise in postmenopausal women [37]. However, this systematic review did not appear to be limited to studies in which participants had been previously participating in exercise levels below that currently recommended for bone health [8]. Consequently, the benefits of exercise could have been underestimated. Another meta-analysis reported a statistically significant benefit of 0.014 g/cm^2^ and 0.012 g/cm^2^, respectively, for both FN and LS BMD in females 60 years of age and older [48]. However, similar to the work of Howe et al. [37], participants did not appear to be limited to those who were participating in exercise levels below that currently recommended for bone health [8]. In addition, all studies were coded by one person, thereby increasing the risk for coding errors [47]. A potential reason for the discrepancy in findings for FN BMD between the Howe et al. [37] and Marques et al. [48] reviews may be accounted for by the fact that the latter meta-analysis limited studies to those in adults 60 years of age and older. This raises the possibility that older postmenopausal women may have more to gain from a regular exercise program. Finally, because the number of analyses aimed at trying to establish the association between selected covariates and changes in FN and LS BMD was limited for all of the previously described meta-analyses, potentially important covariates could have been missed.

A need exists for an updated and thorough meta-analysis on the effects of different ground and joint reaction force exercises, either alone or in combination, on FN and LS BMD in postmenopausal women not participating in exercise levels currently recommended for bone health [8]. Therefore, the purpose of this study was to use the aggregate data meta-analytic approach to determine the effects of ground and/or joint reaction force exercise on BMD at the FN and LS in postmenopausal women not participating in exercise levels currently recommended for bone health [8].

## Methods

### Study eligibility criteria

The *a priori* inclusion criteria for this meta-analysis were as follows: (1) randomized controlled trials, (2) exercise intervention ≥ 24 weeks, (3) comparative control group (attention control, non-intervention, etc.), (4) postmenopausal women, as defined by the authors, (5) participants not currently participating in any type of regular joint and/or ground reaction force exercise, as defined by the authors, (6) published and unpublished (master’s theses and dissertations) studies in any language since January 1, 1989 and (7) BMD (relative value of bone mineral per measured bone area or volume) assessed at the FN and/or LS using dual-energy x-ray absorptiometry (DEXA) or dual-photon absorptiometry (DPA). Given the heterogeneity of reporting by the authors with respect to previous exercise in participants, we revised our inclusion criteria *post hoc* so that only participants who performed less than 150 minutes of moderate intensity (3.0 to 5.9 metabolic equivalents) weight bearing endurance activity per week, less than 75 minutes of vigorous intensity (> 6.0 metabolic equivalents) weight bearing endurance activity per week, resistance training <2 times per week, were included [7]. Studies were limited to those in which exercise was performed for at least 6 months since it has been suggested that one can generally expect exercise-induced changes in BMD to occur after approximately this length of time [55]. Resistance training studies were included only if lower body exercises were part of the exercise program. The year 1989 was chosen as the starting point for the inclusion of studies because it appeared to be the first year in which a randomized controlled intervention trial on exercise and BMD in postmenopausal women was conducted [56]. Studies were limited to those in which BMD at the FN and LS were assessed using either DPA or DEXA since they are/have been the most common instruments for assessing BMD in the clinical setting. Only those groups that met the inclusion criteria were included from each study. Any studies not meeting all of the above criteria were excluded from the meta-analysis.

### Data sources

Studies were retrieved using the following six electronic databases: (1) Medline (within EBSCO host), (2) Embase, (3) Cochrane Central Register of Controlled Trials (CENTRAL), (4) Dissertation Abstracts Online (DAO), (5) CINAHL (within EBSCOhost), and (6) SPORTDiscus (within EBSCOhost). The last search was conducted in August, 2011. All electronic searches were conducted by the second author with assistance from a Health Sciences librarian at West Virginia University. While the search strategies used varied according to the different databases searched, three key words, or forms of keywords, germane to all searches were ‘exercise’, ‘bone’ and ‘randomized’. An example of the search strategy used for one of the electronic database searches (SPORTDiscus) is shown in Additional file 1. In addition to electronic searches, cross-referencing from retrieved studies and previous review articles, both systematic and narrative, was performed. Furthermore, hand searches of selected journals were conducted.

### Study selection

All studies were selected by the first two authors, independent of each other. Disagreements regarding the final list of studies to include were resolved by consensus. If consensus could not be reached, the third author acted as an arbitrator. After an initial list of included studies was developed, the third author reviewed the list for completeness. All included studies as well as a list of excluded studies, including reasons for exclusion, were stored in Reference Manager (version 12.0.1) [57].

### Data abstraction

Prior to data abstraction, a detailed codebook that could hold more than 245 items per study was developed by all three members of the research team in Microsoft Excel 2007 [58]. The major categories of variables that were coded included: (1) study characteristics, (2) subject characteristics, (3) exercise program characteristics, (4) primary outcomes and (5) secondary outcomes. The primary outcomes for this study were BMD at the FN and LS. Secondary outcomes included other measures of BMD (Ward’s triangle, total hip, trochanteric, intertrochanteric, whole body, radius) as well as number of fractures, aerobic fitness, dynamic and static balance, body weight, body mass index (BMI), lean body mass (LBM), fat mass, percent body fat, upper and lower body muscular strength, and calcium and vitamin D intake. Missing primary outcome data were requested from the author(s). Multiple publication bias was avoided by only including data from the most recently published study.

As part of the coding process, the effective load rating for the exercise intervention from each study was calculated using a recently developed, age-adjusted formula [59]. This included the frequency of exercise per week along with the effective load rating, calculated as the product of peak vertical ground reaction force and the rate of force application [59]. Given the multiple types of exercises used in many of the studies, it was not possible to calculate effective load ratings specific to each activity within each study. Therefore, the broad categories recommended by previous work were used [59]. These included numerical effective load ratings equivalent to low (walking, etc.), moderate (tennis, etc.) and high (jumping, etc.) forces [59]. Effective load ratings were also provided for strength training [59]. All studies were coded by the first two authors, independent of each other. They then met and reviewed every entry for accuracy and consistency. Discrepancies were resolved by consensus. If consensus could not be reached, the third author served as an arbitrator.

### Risk of bias

The Cochrane Collaboration risk of bias instrument was used to assess bias across five categories: (1) sequence generation, (2) allocation concealment, (3) blinding to group assignment, (4) incomplete outcome data and (5) incomplete outcome reporting [60]. Each item was classified as having either a high, low, or unclear risk of bias [60]. Assessment for risk of bias was limited to the primary outcomes of interest, i.e. FN and LS BMD. Given the objective nature of BMD assessment, all studies were considered to be at a low risk of bias with respect to blinding unless the study reported some reason for such. For incomplete outcome reporting, studies were considered to be at an unclear risk for bias if studies did not report a study protocol identification number to confirm assessed outcomes. No study was excluded based on the results of the risk of bias assessment [61]. All assessments were performed by the first two authors, independent of each other. Both authors then met and reviewed every item for agreement. Disagreements were resolved by consensus.

### Statistical analysis

#### Calculation of effect sizes for primary and secondary outcomes from each study

Given the different methods of reporting results for primary outcomes, i.e., FN and LS BMD, the standardized mean difference effect size (*g)*, adjusted for small sample bias, was calculated from each study in order to create a common metric for the pooling of findings [62]. Since all studies were parallel, randomized controlled trials [9-33], the *g* for each outcome from each study was calculated as the difference in change scores between the exercise and control groups divided by the pooled SD of the change scores [62]. For studies in which change outcome SDs for the exercise and control groups were not reported, these were estimated for the exercise and control groups using pre-and post-intervention means and SDs according to the approach of Follmann et al. [63]. For studies that did not allow for such calculations using the aforementioned methods, *g* was calculated using the reported 95% confidence intervals (95% CIs). After calculating *g* from each study, its variance was estimated using previously developed procedures [62]. The beneficial effects of exercise on FN and LS BMD were denoted by a positive *g*.

Secondary outcomes from each study were calculated using either *g* (Ward’s triangle, total hip, trochanteric, whole body, radius, calcaneus, aerobic fitness, dynamic and static balance, upper and lower body muscular strength) or the original metric (body weight in kilograms, BMI in kilogram per meters-squared, LBM in kilograms, fat mass in kilograms and percent of body weight, calcium intake in milligrams, vitamin D intake in micrograms).

#### Pooled estimates for FN and LS BMD

Random-effects, method-of-moments models that incorporate heterogeneity into the overall estimate were used to pool results for FN and LS BMD as well as secondary outcomes from each study [64]. Multiple groups from the same study were analyzed independently as well as collapsing multiple groups so that only one ES represented each outcome from each study. For the one study that included both per-protocol and intention-to-treat analyses, the more conservative intention-to-treat results were used [10]. While the same study assessed LS BMD at both the L1-L4 and L2-L4 sites [10], data are reported using the L1-L4 sites based on the International Society for Clinical Densitometry 2007 Position Stand recommending that L1-L4 be used for LS BMD measurement [65]. A *z-score* two-tailed alpha value of ≤0.05 was considered to be statistically significant. Alpha values >0.05 but ≤ 0.10 were considered as a trend. To determine the precision of these estimates, two-tailed 95% confidence intervals (CIs) were also calculated. Analysis of secondary outcomes was considered exploratory because they were not part of the inclusion criteria, and thus, may represent a biased sample.

In terms of magnitude, values for those outcomes in which *g* was used may be classified as either trivial (<0.20), small (≥0.20 to <0.50), medium (≥0.50 to <0.80), or large (≥0.80) [66]. A *g* of 0.20, for example, means that exercise would result in a 0.20 SD benefit over those who did not exercise. Given that the interpretation of *g* can be difficult with respect to clinical and practical relevance [67], the number needed to treat (NNT) was estimated for FN and LS BMD from pooled *g’s* using procedures described by Kraemer and Kupfer [68]. For continuous data, the event is the increase in BMD of magnitude *g.* In addition, the NNT was used to provide a gross estimate of the number of US women 50 years of age and older who could achieve benefit in FN and LS BMD by initiating and maintaining a regular exercise program. This estimate was based on US Census Data for the number of women 50 years of age and older in the US (53,410,602) [69] and Healthy People 2020 Objective PA-2.4 for increasing by 10% the number of adults who meet current physical activity guidelines for aerobic and muscle-strengthening activity [70]. Based on the most recently available physical activity estimates for US adult females, this means an increase in physical activity from 14.9% to approximately 16.4%, a 1.49% increase [71].

#### Stability and validity of changes in *g* for FN and LS BMD

Heterogeneity of results between studies was examined using Q as well as an extension of the Q statistic, *I*^*2*^[72]. Statistical significance for Q was set at an alpha value of ≤0.10. For *I*^*2*^_,_ values of 25% to <50%, 50% to <75%, and ≥75% may be considered to represent small, medium, and large amounts of inconsistency, respectively [72]. To determine treatment effects in a new trial, 95% prediction intervals were also calculated [73,74].

Publication bias was examined using the trim and fill approach of Duval and Tweedie [75]. Potential publication bias was considered noteworthy if a statistically significant finding was no longer significant after imputing potentially missing studies.

In order to examine the effects of each g from each study on the overall findings, results were analyzed with each study deleted from the model once. In addition, standardized residuals ≥ 3.0 were considered as outliers but not arbitrarily deleted from the model. Cumulative meta-analysis, ranked by year, was used to examine the accumulation of evidence over time on FN and LS BMD [76].

#### Moderator analysis for FN and LS BMD

Between-group differences (Q_b_) in FN and LS BMD for categorical variables were examined using mixed effects ANOVA-like models for meta-analysis [77]. This consisted of a random effects model for combining studies within each subgroup and a fixed effect-model across subgroups [77]. Study-to-study variance (tau-squared) was considered not equal for all subgroups. This value was computed within subgroups but not pooled across subgroups. Planned categorical variables to examine *a priori* and in which each category had at least 3 *g’s* included: country in which the study was conducted (USA, other), type of control group (non-intervention, other), matching procedures (yes, no), risk of bias assessment (sequence generation, allocation concealment, blinding, incomplete outcome data, outcome reporting bias according to low, high or unclear risk), type of analysis (per-protocol, intention-to-treat), provision of sample size estimates (yes, no), external funding for the study (yes, no), adverse events (yes, no), whether participants were allowed or required to have osteoporosis, whether they were allowed to be current cigarette smokers and/or consume alcohol (yes, no), changes in exercise habits beyond the exercise intervention (increase, decrease, no change), no prior exercise allowed versus some prior exercise but less than that recommended by the American College of Sports Medicine (yes, no) [8], whether calcium and/or vitamin D supplements were given during the study (yes, no), type of exercise (aerobic, strength, both), exercise delivery (supervised, unsupervised, both), type of reaction forces (ground, joint, both) and instrumentation (Hologic, Lunar). The two-tailed alpha value for a statistically significant difference between groups (Q_b_) was set at *p* ≤ 0.05 with values >0.05 but ≤0.10 considered as a trend. All moderator analyses were considered exploratory [78].

#### Meta-regression for FN and LS BMD

Simple mixed-effects, method of moments meta-regression was used to examine the potential association between changes in FN and LS BMD and continuous variables with at least 3 *g’s*[77]. Because of expected missing data for different variables from different studies, only simple meta-regression was planned and performed. Potential predictor variables, established *a priori,* included year of publication, percentage of dropouts, age in years and years postmenopausal. For exercise training, variables for aerobic-only groups included length (weeks), frequency (days per week), intensity, expressed as a percentage of maximum oxygen consumption (%VO_2max_), percentage of maximal heart rate (MHR) or heart rate reserve (HRR), duration (minutes per session), minutes of training per week and compliance, defined as the percentage of exercise sessions attended. For strength training only groups, variables included: length (weeks), frequency (days per week), intensity, expressed as a percentage of one-repetition maximum (% 1RM), number of sets, repetitions and exercises, rest between sets (seconds) and compliance (%). For those groups that performed both aerobic and strength training concurrently, variables included: length in weeks, frequency (days per week) and percent compliance. Other potential predictors included: load ratings and baseline BMD as well as changes in aerobic fitness, dynamic and static balance, calcium and vitamin D intake, lower and upper body strength, BMI in kg/m^2^, body weight, LBM, percent body fat and fat mass. The alpha value for a statistically significant association was set at ≤0.05. Alpha values >0.05 but ≤0.10 were considered as a trend for an association. All meta-regression analyses were considered exploratory [78].

## Results

### Study characteristics

**Figure 1:**
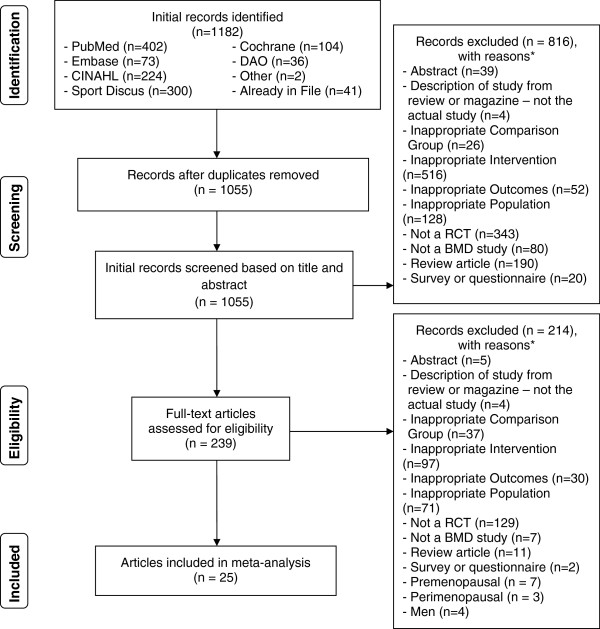
**Flow Diagram for the selection of studies.** *, number of reasons exceeds the number of studies because some studies were excluded for more than one reason.

A general description of the characteristics of each study is shown in Additional file 2. Of the 1,182 citations reviewed, 25 studies representing 63 groups (35 exercise, 28 control) and final assessment of FN and/or LS BMD in 1775 participants, were included [9-33]. One study’s initial exercise inclusion criteria exceeded the exercise eligibility criteria for the current meta-analysis [23]. However, a decision was made to include this study because it was apparent upon further reading that the exercise levels of the participants met the eligibility criteria for the current meta-analysis [23]. Missing primary outcome data were successfully retrieved from three studies [10-12]. The number of exercise participants assessed was 991 while the number of controls assessed was 826. The total (1817) exceeds 1775 because one study had participants exercise one side of the body while the other side served as a control [23]. A description of the search process, including the reasons for excluded studies, is shown in Figure 1. The number of intervention and control groups exceeded the number of studies because some studies included more than one intervention and/or control group that met the inclusion criteria for the current meta-analysis. All studies were published in the English language between the years 1992 and 2011 [9-33]. Twenty-four (96%) were published in journals [9-18,20-33] while one was a dissertation [19]. With respect to country in which the study was conducted, six were performed in the United States [17,18,21,28-30], three in Australia [23,24,31], four in Canada [14,15,25,32], two each in either Brazil [11,12], Japan [20,33], Portugal [26,27], Sweden [10,16], or the United Kingdom [9,13], and one each in China [19] and Germany [22], For types of controls, 11 studies (44%) used a non-intervention control group [9,11,12,16-19,26,27,29,32], while 14 others (56%) used a variety of comparative controls [10,13-15,20-25,28,30,31,33]. Seven of 25 studies (28%) [12,16-19,22,25] reported using the following matching procedures: (1) age [16,22], (2) use of menopausal hormone therapy [12,17], (3) gender [19], (4) BMD and bodyweight [18], (5) postural stability, baseline BMD at the total hip and bisphosphonate use [25]. None of the studies reported using a crossover design. For sample size justification, 12 studies (48%) reported data regarding such [9,10,12,14,16,19,21,22,25-27,30]. Nineteen studies (76%) reported receiving some type of external funding to conduct their study [9,13-17,19,21-31,33].

The dropout rate ranged from 0% to 43% for the 30 exercise groups for which data were available ( $\bar{x}$ ± SD = 17 ± 12%, Mdn = 12%) and 0% to 27% for the 24 control groups in which data were available for ( $\bar{x}$ ± SD = 13 ± 7%, Mdn = 15%). Twelve studies (52%) provided one or more of the following reasons for participants dropping out or for the investigative team to drop participants from the study: (1) personal health problems apparently unrelated to the intervention [13,16,17,26,27,29,30,33], (2) time [14,25,30], (3) lack of compliance to the exercise intervention [10,11], (4) personal issues not related to one’s health [11,13,26,27,33], (5) lack of interest [26] and (6) moved [30]. Five studies (20%) reported that one or more participants experienced musculoskeletal pain and/or minor musculoskeletal injuries as a result of the exercise intervention [9,18,24,29,30]. For the other studies, a lack of complete data were available regarding any possible pain and/or injuries as a result of the interventions. No serious adverse events were reported.

Initial physical characteristics of the participants are shown in Table 1. Fourteen studies (56%) reported data on race/ethnicity with the majority of participants consisting of either whites [14,15,18,21,22,25-30] or Asians [19,20,33]. For medication usage, two studies (8%) included groups in which all participants were taking menopausal hormone therapy [9,17] while four studies (16%) reported that some participants in their groups were taking hormone therapy [12,18,25,30]. One study (4%) reported that some participants were taking bisphophonates [25] while none reported the use of glucocorticoids. With regards to osteoporosis, one study (4%) was limited to participants with osteoporosis [20] while three (12%) reported that some participants had osteoporosis [10,22,25]. Six studies (24%) reported that some participants had osteopenia [10,14,25-27,30]. Ten studies (40%) reported that some participants smoked cigarettes [9,10,13,19,22,25-28,30], while two (8%) reported that some consumed alcohol [15,30]. One study (4%) reported that participants in the exercise intervention group increased their physical exercise outside the intervention while the control group decreased their physical activity [29]. Ten studies (40%) reported giving calcium to participants [10,14,17,20-22,24,28,30,31] whereas another two (8%) provided calcium to some participants [9,29]. Vitamin D was reportedly provided to participants in six studies (24%) [10,14,20-22,28]. A total of three studies (12%) reported that one or more participants had previous fractures [10,25,29].

**Table 1:** Initial physical characteristics of participants

	**Exercise**				**Control**			
**Variable**	**Groups (#)**	$\bar{x}$**±SD**	**Mdn**	**Range**	**Groups (#)**	$\bar{x}$**±SD**	**Mdn**	**Range**
Age (yrs)	33	62.9 ± 7.3	60	54 - 80	27	62.2 ± 6.7	60	53 – 80
Height (cm)	22	161.5 ± 3.3	162	152 - 165	19	161.4 ± 3.2	162	152 – 165
Postmenopausal (yrs)	26	13.8 ± 8	11	3 - 30	21	12.9 ± 7.1	10	4 – 30
Body weight (kg)	28	66.4 ± 6.6	68	46 – 78	23	67.2 ± 7.9	68	46 – 84
Body mass index (kg/m^2^)	21	25.6 ± 2.2	26	20 - 29	18	25.6 ± 2.6	26	20 – 31
Lean body mass (kg)	18	39.2 ± 2.2	39	35 - 43	13	39.1 ± 1.9	39	35 – 42
Fat mass (kg)	6	22.1 ± 5.3	21	17 - 32	4	24.0 ± 8.5	23	15 – 35
Body fat (%)	15	39.3 ± 3.2	39	31 – 44	12	39.1 ± 3.5	39	31 – 46
Calcium intake (mg)	12	846 ± 179	832	609 – 1214	10	868 ± 213	829	626 - 1190
Vitamin D (mcg)	5	5.6 ± 5.1	2	2 - 12	4	5.3 ± 3.9	5	2 – 9
BMD (g/cm^2^)								
- Femoral neck	27	0.749 ± 0.094	0.720	0.580 – 0.925	24	0.766 ± 0.095	0.770	0.590 – 0.927
- Lumbar spine	28	0.957 ± 0.158	0.966	0.595 – 1.180	24	1.00 ± 0.100	1.00	0.600 – 1.200
- Ward’s triangle	8	0.591 ± 0.089	0.575	0.441 – 0.730	6	0.605 ± 0.097	0.598	0.474 – 0.760
- Total hip	13	0.802 ± 0.093	0.840	0.670 – 0.940	11	0.843 ± 0.092	0.869	0.690 – 1.00
- Trochanteric	20	0.659 ± 0.085	0.650	0.510 – 0.806	16	0.682 ± 0.085	0.685	0.520 – 0.840
- Intertrochanteric	11	0.959 ± 0.076	0.986	0.820 – 1.035	7	0.979 ± 0.068	0.990	0.850 – 1.00
- Whole body	8	1.033 ± 0.073	0.99	0.970 – 1.130	7	1.043 ± 0.070	1.002	0.980 – 1.130
- Radius - 1/3	4	0.600 ± 0.028	0.610	0.560 – 0.620	3	0.603 ± 0.012	0.610	0.590 – 0.610
- Radius – mid	4	0.523 ± 0.015	0.530	0.500 – 0.530	3	0.520 ± 0.017	0.530	0.500 – 0.530
- Radius – ultradistal	4	0.363 ± 0.005	0.360	0.360 – 0.370	3	0.363 ± 0.006	0.360	0.360 – 0.370

Notes: Groups (#), number of groups in which data were available; $\bar{x}$±SD, mean ± standard deviation; Mdn, Median; BMD, bone mineral density; Baseline data for aerobic fitness, balance and muscular strength not reported because of the different metrics used in the studies.

Characteristics of the exercise programs from each group and each study are described in Additional file 2. As can be seen, the exercise interventions varied widely. Fourteen groups (40%) participated in exercise interventions that focused on joint reaction forces (for example, strength training) while 12 (34%) focused on ground reaction forces (for example, aerobic exercises such as walking and jumping). Another nine groups (26%) included exercises that provided both joint and ground reaction forces. With the exception of four groups (11%) that performed either jumping or agility training, the remaining 31 (89%) focused on aerobic and/or strength training exercises. The load rating for the 28 groups in which data were available for calculation ranged from 9.4 to 340.5 ( $\bar{x}$ ± SD = 57.3 ± 117.7, Mdn = 10). The length of training across all groups ranged from 24 to 104 weeks ( $\bar{x}$ ± SD = 50.7 ± 23.3, Mdn = 52). A group summary of the characteristics for those studies that included aerobic and/or strength training is shown in Table 2.

**Table 2:** Training program characteristics for aerobic, strength and aerobic + strength training interventions

	**Aerobic**				**Strength**				**Aerobic + Strength**			
**Variable**	**Groups (#)**	$\bar{x}$**±SD**	**Mdn**	**Range**	**Groups (#)**	$\bar{x}$**±SD**	**Mdn**	**Range**	**Groups (#)**	$\bar{x}$**±SD**	**Mdn**	**Range**
Length (weeks)	9	52 ± 22	52	24-104	14	46 ± 21	52	24-104	10	58 ± 29	52	24-104
Frequency (days/week)	8	3 ± 1	3	3-4	14	3 ± 1	3	3-6	9	3 ± 1	3	2-7
Intensity*	4	55 ± 14	59	36-68	6	63 ± 26	73	15-85	-	-	-	-
Duration (min/sessions)	6	34 ± 12	38	10-30	-	-	-	-	-	-	-	-
Minutes (per week)	6	103 ± 37	113	60-135	-	-	-	-	-	-	-	-
Minutes (per week adjusted)	5	79 ± 33	71	48-113	-	-	-	-	-	-	-	-
Sets (#)	NA	NA	NA	NA	12	3 ± 1	3	1-5	5	2 ± 0.4	2	2-3
Repetitions (#)	NA	NA	NA	NA	9	12 ± 8	10	8-30	-	-	-	-
Rest between sets (sec.)	NA	NA	NA	NA	4	75 ± 57	90	0-120	-	-	-	-
Exercises (#)	NA	NA	NA	NA	14	8 ± 4	9	1-12	5	8 ± 3	7	4-12
Compliance (%)	7	75 ± 16	80	39-84	10	83 ± 5	85	74-90	7	76 ± 11	77	59-95

Groups (#), number of groups in which data were available; $\bar{x}$±SD, mean ± standard deviation; Mdn, Median; *, intensity expressed as a percentage of maximum oxygen consumption for aerobic groups and percentage of one-repetition maximum for strength training groups; -, insufficient data to calculate; NA, not applicable.

Bone mineral density assessment information is shown in Additional file 2. With the exception of two earlier studies that used dual photon absorptiometry [18,28], all others used dual-energy x-ray absorptiometry to assess BMD at the FN and LS [9-17,19-27,29-33]. The two most common instruments used to assess FN and LS BMD were Hologic (48%) and Lunar (40%). For those studies that provided data [9,13,14,16,20,22-27,30,32], coefficients of variation for the assessment of BMD ranged from 0.8% to 1.9% and 0.6% to 1.5%, respectively, for FN and LS BMD.

### Risk of bias assessment

**Figure 2:**
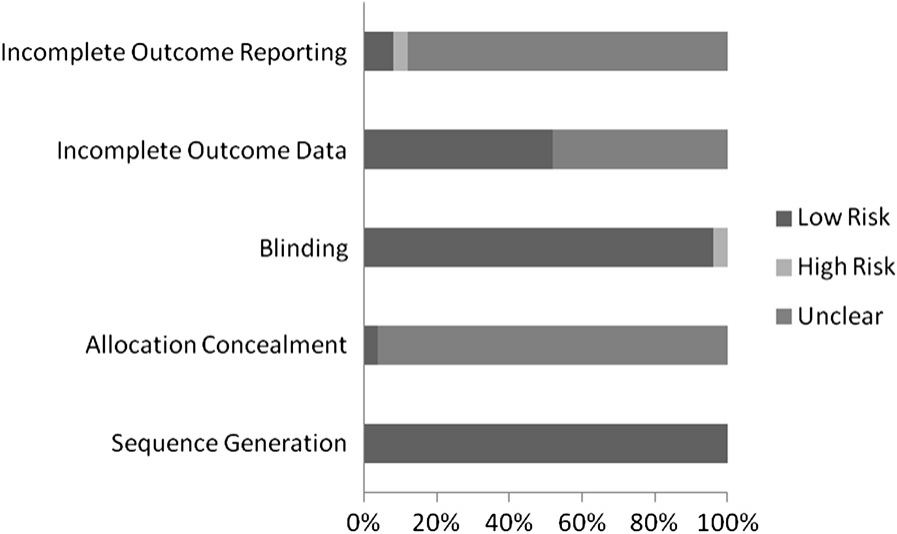
**Risk of bias.** Pooled risk of bias results using the Cochrane Risk of Bias Assessment Tool [60].

Risk of bias results are shown in Figure 2. As can be seen, the majority of studies were considered to be at low risk with respect to sequence generation and blinding and unclear risk for allocation concealment and incomplete outcome reporting. Approximately half of the studies were considered to be at either low or unclear risk for incomplete outcome data.

### Primary outcomes

#### FN BMD

**Figure 3:**
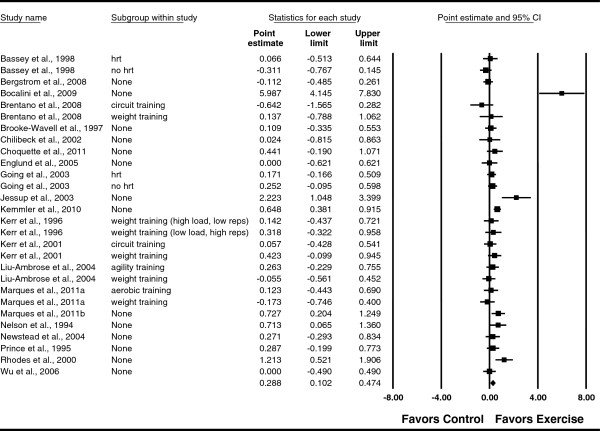
**Forest plot for changes in FN BMD.** Forest plot for point estimate standardized effect size changes (*g*) in FN BMD. The black squares represent the standardized mean difference (*g*) while the left and right extremes of the squares represent the corresponding 95% confidence intervals. The middle of the black diamond represents the overall standardized mean difference (*g*) while the left and right extremes of the diamond represent the corresponding 95% confidence intervals. For subgroup, HRT means hormone replacement therapy.

**Figure 4:**
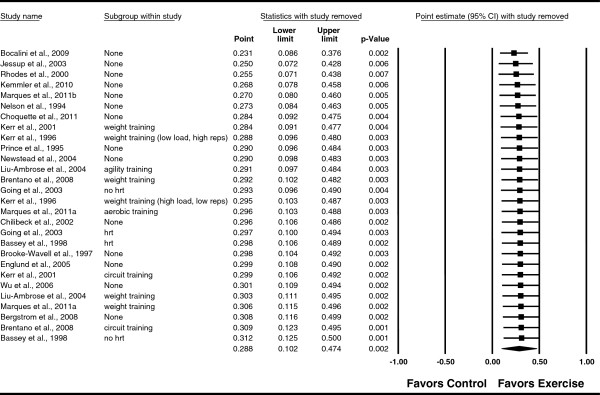
**Influence analysis for changes in FN BMD.** Influence analysis for point estimate standardized effect size changes (*g*) in FN BMD with each corresponding study deleted from the model once. The black squares represent the standardized mean difference (*g*) while the left and right extremes of the squares represent the corresponding 95% confidence intervals. The middle of the black diamond represents the overall standardized mean difference (*g*) while the left and right extremes of the diamond represent the corresponding 95% confidence intervals. Results are ordered from smallest to largest values of *g.* For subgroup, HRT means hormone replacement therapy.

**Figure 5:**
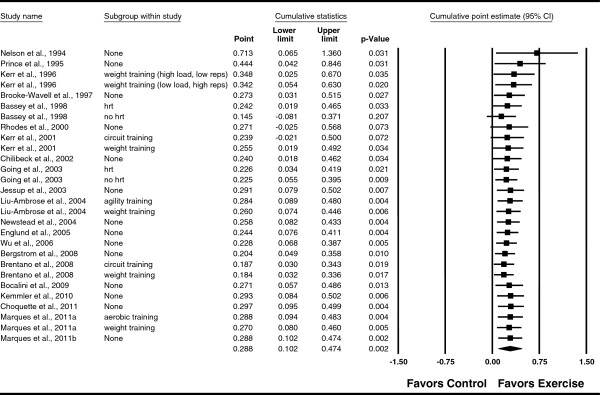
**Cumulative meta-analysis for changes in FN BMD.** Cumulative meta-analysis, ordered by year, for point estimate standardized effect size changes (*g*) in FN BMD. The black squares represent the standardized mean difference (*g*) while the left and right extremes of the squares represent the corresponding 95% confidence intervals. The results of each corresponding study are pooled with all studies preceding it. The middle of the black diamond represents the overall standardized mean difference (*g*) while the left and right extremes of the diamond represent the corresponding 95% confidence intervals. For subgroup, HRT means hormone replacement therapy.

Overall, there was a statistically significant benefit of ground and/or joint reaction force exercise on FN BMD (Table 3, Figure 3). In addition, non-overlapping CIs were observed. The NNT was 6 with an estimated 127,968 postmenopausal US women experiencing benefit in FN BMD if they began and maintained a regular exercise program. A moderate but statistically significant amount of heterogeneity was observed as well as overlapping prediction intervals. No adjustment for publication bias was needed. With each study deleted from the model once, results remained statistically significant across all deletions (Figure 4). The difference in *g* between the largest and smallest values with each study deleted from the model was 0.081 (26.0%). With two outliers removed [11,21], results remained statistically significant (*g* = 0.207, 95% CI = 0.076, 0.338, *p* = 0.002) and heterogeneity, while statistically significant (Q = 42.2, *p* = 0.02), was reduced to 40.7%. Improvements in FN BMD also remained statistically significant when data were collapsed so that only one *g* represented each study (*g* = 0.343, 95% CI = 0.129, 0.556, *p* = 0.002, Q = 85.5, *p* <0.0001, *I*^*2*^ = 76.6%). Cumulative meta-analysis, ranked by year, demonstrated that results have been statistically significant, or there has been a trend for statistical significance, since 2000 (Figure 5).

**Table 3:** Changes in primary and secondary outcomes

**Variable**^**a**^	**Studies (#)**	**ES (#)**	**Participants (#)**	$\bar{x}$**(95% CI)**	***Z*(*p*)**	**Q(*p*)**	***I***^***2 ***^***(%)***	**95% PI**
Primary								
- Femoral neck	21	28	1632	0.288 (0.102, 0.474)	3.03(0.002)*	90.5(*p* <0.0001)*	70.1	−0.568, 1.142
- Lumbar spine	21	28	1504	0.179 (−0.003, 0.361)	1.93(0.05)*	77.7(<0.0001)*	65.3	−0.614, 0.972
Secondary								
- Ward’s triangle	6	8	252	0.260 (−0.405, 0.613)	0.40(0.69)	28.1(<0.0001)*	75.1	−1.567, 1.775
- Total hip	10	14	734	0.232 (0.073, 0.391)	2.86(0.004)*	17.6(0.18)	26.0	−0.149, 0.613
- Trochanteric	14	21	1085	0.222 (0.107, 0.337)	3.79(<0.0001)*	18.3(0.57)	0	0.099, 0.345
- Intetrochanteric	6	10	399	0.241 (0.058, 0.425)	2.58(0.01)*	8.3(0.50)	0	0.024, 0.458
- Whole body	6	7	246	0.121 (−0.055, 0.298)	1.35(0.18)	2.7(0.85)	0	−0.110, 0.352
- Radius - 1/3	2	4	182	0.048 (−0.329, 0.424)	0.25(0.81)	5.8(0.12)	48.2	−1.365, 1.461
- Radius – mid	2	4	182	0.153 (−0.262, 0.568)	0.72(0.47)	7.0(0.07)**	57.2	−1.496, 1.802
- Radius – ultradistal	2	4	182	0.263 (−0.239, 0.765)	1.03(0.31)	10.1(0.02)*	70.3	−1.886, 2.412
- Aerobic fitness	5	8	198	1.146 (0.31, 1.930)	2.86(0.004)*	47.0(*p* <0.0001)*	85.1	−1.539, 3.831
- Dynamic balance	4	5	95	1.39 (0.766, 2.014)	4.37(<0.0001)*	18.9(0.001)*	78.9	−0.856, 3.636
- Static balance	5	7	112	0.841 (0.228, 1.454)	2.69(0.007)*	40.9(<0.0001)*	85.3	−1.254, 2.936
- Body weight (kg)	11	17	594	−0.03 (−0.4, 0.4)	−0.15(0.88)	13.0(0.67)	0	−0.5, 0.4
- Body mass index (kg/m^2^)	8	11	511	−0.2 (−0.8, 0.4)	−0.69(0.49)	109.9(<0.0001)*	90.9	−2.3, 1.9
- Lean body mass (kg)	7	10	461	0.4 (−0.06, 0.9)	1.72(0.09)**	23.8(0.005)*	62.1	−1.0, 1.9
- Fat mass (kg)	4	6	230	−0.5 (−1.2, 0.2)	−1.48(0.14)	11.0(0.05)*	54.6	−2.4, 1.4
- Body fat (%)	5	7	211	−1.7 (−2.8, -0.8)	−3.58(<0.0001)*	13.1(0.04)*	54.1	−4.4, 0.8
- Strength (upper body)	7	9	300	2.01 (1.08, 2.95)	4.24(<0.0001)*	97.8(<0.0001)*	97.8	−1.33, 5.36
- Strength (lower body)	9	12	482	1.58 (0.91, 2.24)	4.67(<0.0001)*	120.9(<0.0001)*	90.9	−1.00, 4.10
- Calcium intake (mg)	5	7	319	10.1 (−15.8, 35.9)	0.76(0.45)	0.3(1.0)	0	−23.9, 44.0
- Vitamin D (mcg)	--	--	--	--	--	--	--	--

Notes: ^a^Unless noted otherwise, all outcomes are reported as standardized effect size (g); ES, effect size; #, number; Z(*p*), *z-*score and alpha value; Q(*p*), Cochran’s Q statistic and alpha value; *I*^*2 *^*(%),* I-squared; PI, prediction intervals. *, statistically significant (*p* ≤ 0.05; **trend for statistical significance (*p* >0.05 to ≤ 0.10); --, Insufficient data reported (< 3 ES’s).

Moderator analysis for changes in FN BMD is shown in Additional file 3. As can be seen, no statistically significant between-group differences (Q_b_) were found for those *a priori* comparisons for which sufficient data were available.

Meta-regression analyses for changes in FN BMD are shown in Additional file 4. As can be seen, there was a statistically significant association between increases in FN BMD and decreased compliance (combined aerobic and strength training groups only), decreases in BMI, decreases in body weight and decreases in percent body fat. A trend for a statistically significant association was observed for increases in FN BMD and increases in intensity (strength only), increased compliance (strength training group only) and increases in static balance.

#### LS BMD

**Figure 6:**
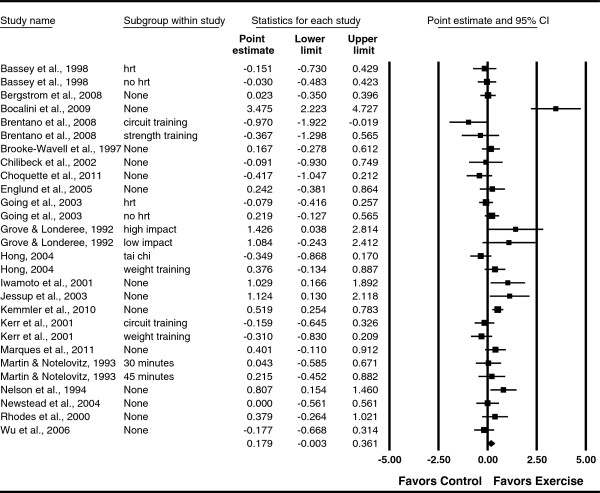
**Forest plot for changes in LS BMD.** Forest plot for point estimate standardized effect size changes (*g*) in LS BMD. The black squares represent the standardized mean difference (*g*) while the left and right extremes of the squares represent the corresponding 95% confidence intervals. The middle of the black diamond represents the overall standardized mean difference (*g*) while the left and right extremes of the diamond represent the corresponding 95% confidence intervals. For subgroup, HRT means hormone replacement therapy.

**Figure 7:**
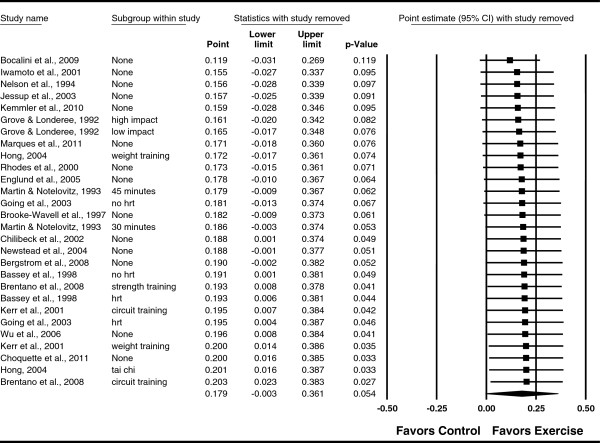
**Influence analysis for changes in LS BMD.** Influence analysis for point estimate standardized effect size changes (*g*) in LS BMD with each corresponding study deleted from the model once. The black squares represent the standardized mean difference (*g*) while the left and right extremes of the squares represent the corresponding 95% confidence intervals. The middle of the black diamond represents the overall standardized mean difference (*g*) while the left and right extremes of the diamond represent the corresponding 95% confidence intervals. Results are ordered from smallest to largest values of *g.* For subgroup, HRT means hormone replacement therapy.

**Figure 8:**
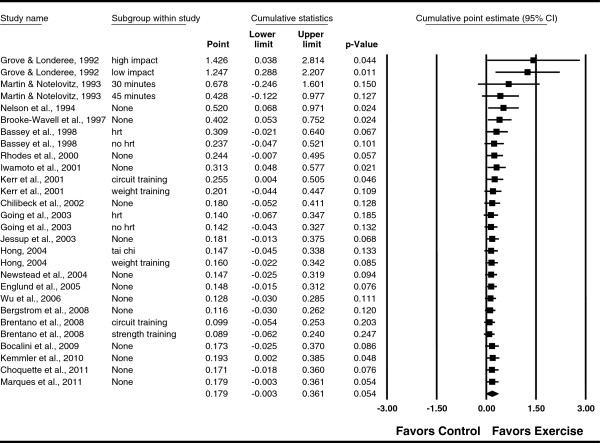
**Cumulative meta-analysis for changes in LS BMD.** Cumulative meta-analysis, ordered by year, for point estimate standardized effect size changes (*g*) in LS BMD. The black squares represent the standardized mean difference (*g*) while the left and right extremes of the squares represent the corresponding 95% confidence intervals. The results of each corresponding study are pooled with all studies preceding it. The middle of the black diamond represents the overall standardized mean difference (*g*) while the left and right extremes of the diamond represent the corresponding 95% confidence intervals. For subgroup, HRT means hormone replacement therapy.

Overall, there was a statistically significant benefit in LS BMD but slightly overlapping 95% CIs (Table 3, Figure 6). The NNT was 6 with an estimated 80,219 postmenopausal US women maintaining and/or increasing their LS BMD if they began and maintained a regular exercise program. A moderate and statistically significant amount of heterogeneity was observed as well as overlapping prediction intervals. No adjustment for publication bias was needed. With the exception of one study [11], an outlier, results remained statistically significant or there was a trend for statistical significance when each study was deleted from the model once (Figure 7). The difference in *g* between the largest and smallest values was 0.084 (41%) when each study was deleted. With the one outlier deleted from the model, the alpha value for *g* increased to 0.12 and heterogeneity, while still statistically significant (Q = 42.2, *p* = 0.02), was reduced to 48.5%. The benefits in LS BMD remained statistically significant when data were collapsed so that only one *g* represented each study (g = 0.231, 95% CI = 0.026, 0.435, *p* = 0.03, Q = 71.1, *p* <0.0001, *I*^*2*^ = 71.9%). Cumulative meta-analysis, ranked by year, demonstrated that results have been statistically significant, or there has been a trend for statistical significance, since 2009 (Figure 8).

Moderator analysis for changes in LS BMD is shown in Additional file 3. As can be seen, no statistically significant between-group differences (Q_b_) were found for those *a priori* comparisons in which sufficient data were available.

Meta-regression analyses for changes in LS BMD are shown in Additional file 4. As shown, there was a statistically significant association between increases in LS BMD and older age, greater number of years postmenopausal, fewer minutes of training per session (aerobic groups only), fewer minutes of training per week, greater intensity of training (strength only), increased compliance (strength only), decreased compliance (combined aerobic and strength training only), increases in static balance, decreases in BMI, body weight and percent body fat. A trend for a statistically significant association was found between increases in LS BMD and smaller increases in aerobic fitness as well as increases in lean body mass.

### Secondary outcomes

Changes in secondary outcomes are shown in Table 3. As can be seen there was a statistically significant benefit in BMD at the total hip, trochanteric and intertrochanteric regions. A non-significant and small to nil amount of heterogeneity was observed for all three outcomes. In addition, non-overlapping prediction intervals were observed for the trochanteric region. Furthermore, large, statistically significant improvements as well as statistically significant and large amounts of heterogeneity were found for aerobic fitness, dynamic and static balance. For body composition, a trend for statistically significant increases in LBM along with a statistically significant and moderate amount of heterogeneity was observed. A statistically significant decrease as well as a statistically significant and moderate amount of heterogeneity was also observed for percent body fat. For both upper and lower body strength, large, statistically significant increases were observed as well as large and statistically significant amounts of heterogeneity. Insufficient data were available to examine differences in fractures between the exercise and control groups.

## Discussion

The purpose of this study was to use the aggregate data meta-analytic approach to determine the effects of ground and/or joint reaction force exercise on BMD at the FN and LS in postmenopausal women participating in exercise levels below that currently recommended for bone health [8]. The overall results suggest that ground and joint reaction force exercise may result in clinically important benefits in FN and LS BMD, with results more convincing for FN BMD. These findings are similar to those from three [48,51,53] of four [37,48,51,53] previous meta-analyses for FN BMD and four [37,39,48,53] of five [37,39,48,51,53] previous meta-analyses for LS BMD, all of which included both ground and joint reaction force exercises from randomized controlled trials in postmenopausal women. Further support for the overall findings of the current meta-analysis were strengthened by the robustness of results when data were collapsed so that only one *g* represented each study as well as when examined for publication bias. When each study was deleted from the model once, results remained statistically significant for FN BMD across all deletions but were no longer statistically significant for LS BMD (*p* = 0.12) when one study was deleted from the model [11]. From a stability perspective, the statistical significance of findings has been consistent over a longer period of time for BMD at the FN (2000) versus LS (2009). Thus, the changes in BMD appear to be more convincing for FN versus LS BMD. This may have to do with the possibility that the exercise protocols employed were more specific to the FN versus LS.

While random-effects models that incorporate heterogeneity into the analysis were used, it is still important to point out that heterogeneity was observed for both FN and LS BMD. The existence of heterogeneity in meta-analysis is not only common [79], but also important, as there is no need to combine studies exactly alike since their findings, within statistical error, would be the same [80]. In addition, prediction intervals for estimating the expected results of a new trial included zero for both FN and LS BMD. However, these values should not be confused with confidence intervals since prediction intervals are based on a random mean effect while confidence intervals are not [73]. Nevertheless, these prediction intervals may be beneficial for future researchers interested in conducting randomized controlled intervention trials addressing the effects of ground and/or joint reaction force exercise on FN and LS BMD in postmenopausal women.

While the magnitude of change in FN and LS BMD might be considered small at the FN and trivial at the LS, they appear to be clinically important. For example, based on previous prediction models [81], the exercise-induced changes in BMD observed at the FN and LS in the current meta-analysis would reduce the 20-year relative risk of osteoporotic fracture at any site by approximately 11% and 10%, respectively. However, the observed benefits of exercise on FN (g = 0.29) and LS (g = 0.18) BMD in the current meta-analysis were smaller than those previously reported for pharmacologic interventions (alendronate, calcitonin, etidronate, hormone therapy, raloxifine, risedronate) at both the hip (range of *g* = 0.64 to 5.74) and LS (range of *g* = 0.90 to 8.90) [82]. The exercise-induced benefits on FN and LS BMD also appear to be similar to or smaller than those observed for calcium and vitamin D supplementation (*g* for calcium = 0.45 at the hip and 1.57 at the LS; *g* for vitamin D = 0.47 at the hip and 0.20 at the LS) [82]. However, the use of pharmacological and nutritional interventions should be considered with respect to several factors. These include: (1) the potential adverse effects of pharmacologic agents [83], (2) that participants included in previous pharmacological and nutritional intervention studies had generally lower initial levels of BMD than participants included in the current exercise meta-analysis [83], and (3) that exercise results in numerous other benefits not realized with pharmacologic and nutritional interventions [84], for example, increases in balance and a subsequent reduction in falls [85]. Given the former, the current recommendations of lifestyle changes such as exercise and adequate calcium and vitamin D intake prior to pharmacological intervention appear to be appropriate [6].

The focus of the present meta-analysis has been on the use of the traditional alpha value for statistical significance (p ≤ 0.05) and 95% CI. However, it has been suggested that rather than focus on the term statistically significant and alpha value cutpoints, one should report the exact alpha value and use 90% CI to determine clinical relevance within the range of the 90% interval [86]. Using the 90% CI approach, the interval no longer included zero (0) for changes in LS BMD (0.026 to 0.332) and ranged from 0.132 to 0.444 for changes in FN BMD.

No statistically significant between-group differences were found when mixed-effects ANOVA was conducted for changes in FN and LS BMD partitioned by a large number of categorical variables. However, while no statistically significant between-group differences were noted, changes in FN BMD were smaller for ground (*g =* 0.088) versus joint (*g* = 0.420) and combined joint and ground reaction force exercise (*g* = 0.398).

Several interesting associations were found when simple meta-regression was performed for changes in FN and LS BMD. For ease of reading, statistically significant findings (*p* < 0.05) as well as trends for statistical significance (>0.05 but ≤ 0.10) are discussed collectively. For both FN and LS BMD, greater increases were associated with both greater intensity and compliance in the strength training (joint-reaction force) groups. These findings suggest that greater loads per repetition as well as greater adherence may provide greater benefit to FN and LS BMD. Greater improvements in both FN and LS BMD were also associated with increases in static balance. These associations may be especially important for reducing the risk of falling as well as subsequent fracture risk. Greater increases in both FN and LS BMD were also associated with decreases in BMI, body weight and percent body fat. In addition, increases in LS BMD were associated with increases in LBM. All of these associations may be reflective of greater exercise effort. The inverse association between increases in both FN and LS BMD with poorer compliance to aerobic and strength training protocols may be nothing more than the play of chance. Alternatively, studies with poorer compliance may have yielded greater benefits in FN and LS BMD because of the greater overall volume of training prescribed. For LS BMD, the positive association between increases in LS BMD and older age as well as a greater number of years postmenopausal may be the result of lower initial levels of BMD. However, we found no association between baseline LS BMD and changes in LS BMD. The negative associations between increases in LS BMD with shorter duration and total minutes of training per week for aerobic exercise studies may help to reinforce the belief that shorter duration activities such as jumping may be more beneficial to LS BMD than activities such as walking [7]. One potential reason for this negative association may be the result of calcium loss from excessive sweating in longer duration and/or higher intensity activities [87,88]. This causes a decrease in serum calcium followed by an increase in serum parathyroid hormone, which then stimulates bone resorption [87,88]. While these findings are interesting, further research is needed before any firm conclusions can be drawn.

In addition to changes in FN and LS BMD, statistically significant improvements were found for several secondary outcomes. These included increases in BMD (total hip, trochanteric, intertrochanteric), aerobic fitness, dynamic and static balance, lean body mass and both upper and lower body strength. Statistically significant decreases in percent body fat were also found. These findings reinforce the many benefits that can be derived from exercise programs [84]. The former notwithstanding, the results for secondary outcomes should be interpreted with caution since they were only included if FN and/or LS BMD data were reported. Consequently, secondary outcomes in meta-analysis may not comprise a representative sample.

A major interest of the investigative team was to examine the dose–response relationship between changes in FN and LS BMD and exercise load ratings in postmenopausal women. While we found no significant association between changes in FN and LS BMD and load ratings, these associations were based on general categorical estimates versus estimates specific to each activity [59]. The decision to use categorical estimates was based on the inability to accurately calculate load ratings for those studies that involved multiple types of activities. In addition, the algorithm used requires further testing, improvement and validation [59]. Future research should also focus on developing formulas for accurately calculating load ratings from data typically provided in randomized controlled intervention trials. Ideally, individual studies should collect and report force data in all exercise interventions. However, the accurate measurement of such may be challenging for some activities [7]. Until additional dose–response research is conducted, it would appear plausible to suggest that postmenopausal women adhere to the exercise guidelines from the American College of Sports Medicine [8]. These include weight-bearing endurance activities 3 to 5 times per week as well as resistance exercise 2 to 3 times per week [8]. However, it will be particularly important for future dose–response studies to determine whether increased duration of aerobic exercise diminishes the potential skeletal benefits, as suggested by the current regression analyses.

The results of this meta-analysis should be viewed with respect to several potential limitations. First, because studies are not randomly assigned to covariates, they are considered to be observational in nature. Therefore, the results of moderator and regression analyses conducted in this or any other meta-analysis do not support causal inferences [78]. Second, because a large number of statistical tests were conducted, some statistically significant results could have been nothing more than the play of chance. However, as suggested by Rothman [89], no adjustment was made for multiple tests because of the concern about missing possibly important findings. Third, because of a lack of data, a common occurrence in meta-analysis, the research team was unable to examine several variables, thereby compromising the thoroughness of the study. With the former in mind, it is suggested that future randomized controlled trials addressing the effects of ground and/or joint reaction force exercise on FN and LS BMD in postmenopausal women include information regarding study design (allocation concealment, incomplete outcome data, verification that all outcomes planned to be assessed are reported), participant characteristics (adverse events, whether the participants had osteoporosis, cigarette smoking, alcohol consumption, change in exercise habits outside the intervention) and exercise intervention characteristics (intensity, how exercise was delivered). Fourth, future studies should provide more specific information regarding their exercise cutpoints for enrolling participants in their studies. The heterogeneity of reporting found in the current meta-analysis is not surprising. In a systematic review of the different definitions of sedentary for screening participants for entrance into physical activity intervention trials, Bennett et al. [90], found that the definition of sedentary ranged from less than 20 to less than 150 minutes per week minutes of physical activity and that few studies reported the type and intensity of physical activity used to screen participants. While such varied definitions may make it difficult to generalize findings, the current meta-analysis, to the best of the authors’ knowledge, is the first one on exercise and BMD in women to limit the inclusion of studies to those in which participants were not currently meeting exercise recommendations for bone health [8]. Fifth, given the potential advantage of high resolution peripheral quantitative computed tomography (HR-pQCT) for detecting microarchitectural changes in bone [91], it would appear plausible to suggest that future exercise intervention studies should use this technology so as to better understand the exercise-induced changes that may occur in bone. Finally, consistent with recommendations from the 2008 Physical Activity Guidelines Report, there continues to be a need for large randomized controlled trials to determine whether fracture incidence is decreased as a result of ground and/or joint reaction force exercise [7].

## Conclusions

The overall findings of this aggregate data meta-analysis suggest that exercise may result in clinically relevant benefits to FN and LS BMD in postmenopausal women. Several observed and important associations appear worthy of further investigation in well-designed randomized controlled trials.

## Competing interests

The authors declare that they have no competing interests.

## Authors’ contributions

GAK was responsible for the conception and design, acquisition of data, analysis and interpretation of data, drafting the initial manuscript and revising it critically for important intellectual content. KSK was responsible for the conception and design, acquisition of data, and reviewing all drafts of the manuscript. WMK was responsible for the conception and design, interpretation of data and reviewing all drafts of the manuscript. All authors read and approved the final manuscript.

## Authors’ information

GAK has more than 15 years of successful experience in the design and conduct of all aspects of meta-analysis, including the effects of chronic exercise on bone mineral density in adult humans. KSK has more than 12 years of successful experience in conducting meta-analysis, including the effects of chronic exercise on bone mineral density in adult humans. WMK is a leading authority on the effects of exercise on bone mineral density.

## Pre-publication history

The pre-publication history for this paper can be accessed here:

http://www.biomedcentral.com/1471-2474/13/177/prepub

## Supplementary Material

Additional file 1 **Example of search strategy for one database search (SPORTDiscus).** This additional file describes the search strategy used searching the SPORTDiscus database for randomized controlled trials dealing with the effects of exercise on bone mineral density in adults. Click here for file

Additional file 2 **General characteristics of included studies.** This additional file provides a description of the general characteristics of studies that met the inclusion criteria for the meta-analysis. Click here for file

Additional file 3 **Table of moderator analyses results for FN and LS BMD.** This additional file provides a table of results for all moderator analyses that were conducted for categorical variables and changes in femoral neck and lumbar spine bone mineral density. Click here for file

Additional file 4 **Table of meta-regression results for changes in FN and LS BMD.** This additional file provides a table of results for all regression analyses that were conducted for changes in femoral neck and lumbar spine bone mineral density. Click here for file
