# Supplementary material for: Effects of ground and joint reaction force exercise on lumbar spine and femoral neck bone mineral density in postmenopausal women: a meta-analysis of randomized controlled trials
Source: BMC Musculoskelet Disord. 2012 Sep 20;13:177. doi: 10.1186/1471-2474-13-177 (PMC3489866; doi:10.1186/1471-2474-13-177)
Supplement: Additional file 1 — Example of search strategy for one database search (SPORTDiscus). This additional file describes the search strategy used searching the SPORTDiscus database for randomized controlled trials dealing with the effects of exercise on bone mineral density in adults. [file 1471-2474-13-177-S1.doc]

Additional File 1. Example of search strategy for one database search (SPORTDiscus)

EBSCOhost: Print Search History

Print Search History Friday, June 18, 2010 10:08:45 AM

# Query Limiters/Expanders Last Run Via Results

S11 (s7 and s10) Limiters - Published Date: 19890101-20100631

Search modes - Find all my search terms Interface - EBSCOhost

Search Screen - Advanced Search

Database - SPORTDiscus with Full Text 300

S10 (s8 or s9) Limiters - Published Date: 19890101-20100631

Search modes - Find all my search terms Interface - EBSCOhost

Search Screen - Advanced Search

Database - SPORTDiscus with Full Text 135179

S9 (teenager* or adolescen* or teen* or adult or senior or aged or

geriatric or geriatrics or elder or elderly) Limiters - Published Date:

19890101-20100631

Search modes - Find all my search terms Interface - EBSCOhost

Search Screen - Advanced Search

Database - SPORTDiscus with Full Text 84621

S8 human Limiters - Published Date: 19890101-20100631

Search modes - Find all my search terms Interface - EBSCOhost

Search Screen - Advanced Search

Database - SPORTDiscus with Full Text 59183

S7 (s3 and s6) Limiters - Published Date: 19890101-20100631

Search modes - Find all my search terms Interface - EBSCOhost

Search Screen - Advanced Search

Database - SPORTDiscus with Full Text 639

S6 (s4 or s5) Limiters - Published Date: 19890101-20100631

Search modes - Find all my search terms Interface - EBSCOhost

Search Screen - Advanced Search

Database - SPORTDiscus with Full Text 23853

S5 TX clinical w1 trial* Limiters - Published Date: 19890101-20100631

Search modes - Find all my search terms Interface - EBSCOhost

Search Screen - Advanced Search

Database - SPORTDiscus with Full Text 16823

S4 TX random* w1 control* Limiters - Published Date: 19890101-20100631

Search modes - Find all my search terms Interface - EBSCOhost

Search Screen - Advanced Search

Database - SPORTDiscus with Full Text 13437

S3 (s1 and s2) Limiters - Published Date: 19890101-20100631

Search modes - Find all my search terms Interface - EBSCOhost

Search Screen - Advanced Search

Database - SPORTDiscus with Full Text Display

S2 (MH "bone density") or TX bone w1 densit* Limiters - Published Date:

19890101-20100631

Search modes - Find all my search terms Interface - EBSCOhost

Search Screen - Advanced Search

Database - SPORTDiscus with Full Text Display

S1 TX exercise or MH exercise Limiters - Published Date: 19890101-20100631
