# Supplementary material for: Effects of ground and joint reaction force exercise on lumbar spine and femoral neck bone mineral density in postmenopausal women: a meta-analysis of randomized controlled trials
Source: BMC Musculoskelet Disord. 2012 Sep 20;13:177. doi: 10.1186/1471-2474-13-177 (PMC3489866; doi:10.1186/1471-2474-13-177)
Supplement: Additional file 2 — General characteristics of included studies. This additional file provides a description of the general characteristics of studies that met the inclusion criteria for the meta-analysis. [file 1471-2474-13-177-S2.doc]

Additional File 2. General characteristics of included studies.

| Study | Country | Participants | Exercise Intervention | BMD Assessment |
| --- | --- | --- | --- | --- |
| Bassey et al., 1998[9] | United Kingdom | 123 healthy, postmenopausal women assigned to an exercise + no HT (n=45, age, 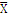 ± SD= 55.8 + 3.3 yrs), exercise + HT (n=24, age, 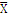 ± SD= 53.7 + 3.2 yrs), no HT, no exercise control (n=32, age, 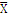 ± SD= 54.9 + 4.1 yrs) or HT control (n=22, age, 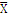 ± SD= 53.4 + 4.5 yrs) group  No current or recent (12 months) participation in vigorous, regular exercise more than 1h/wk | 50 jumps/day (10-min session consisting of stretching plus 5 bouts of 10 vertical jumps), 6 days/ wk for 51 wks | DEXA (Lunar DPX-L) at the LS & FN |
| Bergstrom et al., 2008[10] | Sweden | 112 postmenopausal women 45 to 65 yrs of age assigned to either a physical training (n=60) or control (n=52) group  Not already training at the level of or above that of the intervention | 3 fast 30-min walks and 1 to 2 sessions of 1h training (5 min warm-up, 25 min strengthening exercise for arms, legs, back & stomach, 25 min aerobic exercise, 5 min stretching) per wk for 1 yr | DEXA (GE Medical Systems Lunar Prodigy 10631) at the LS & FN |
| Bocalini et al., 2009[11] | Brazil | 25 postmenopausal women assigned to either a trained (n=15, age, 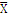 ± SD= 69 + 34.86 yrs) or an untrained control (n=10, age, 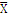 ± SD= 67 + 25.29 yrs) group  No participation in a regular and structured physical activity for the last 3 months | 3 resistive exercise sessions/wk, 1 h/session, 12 exercises, 3 sets of 10 reps at 85% 1RM, for 24 wks | DEXA (Osteometer MediTech DEA-DTX 200) at the LS & FN |
| Brentano et al., 2008[12] | Brazil | 28 postmenopausal women assigned to a strength training (n=9), circuit training (n=10), or control (n=9) group  Not engaged in any type of regular exercise | 3 days/wk, 1 h/session, 10 exercises, Circuit: 2-3 sets, 10-20 reps, 45-60% 1RM; Strength: 2-4 sets, 6-20 reps, 45-80% 1RM, for 24 wks | DEXA (Hologic QDR 4500A) at the LS & FN |
| Brooke-Wavell et al.,1997[13] | United Kingdom | 79 healthy, postmenopausal women 60 to 70 yrs of age assigned to either a walking (n=39) or control (n=40) group  No participation in regular exercise | Brisk walking for 280 min/wk, each session 20-50 min, 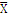 ± SD= 20.4 + 3.8 min/day, for 1 yr | DEXA (Lunar DPX-L) at the LS & FN |
| Chilibeck et al., 2002[14] | Canada | 22 postmenopausal women assigned to either an exercise+placebo (n=10, 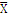 ± SD= 56.8 + 2 yrs of age) or placebo (n=12, 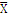 ± SD= 58.8 + 1.8 yrs of age) group  No recent participation in vigorous exercise | Strength training, 3 days/wk, 12 exercises, 2 sets, 8-10 reps, 70% 1RM, for 1 yr | DEXA (Hologic QDR-2000) at the LS & FN |
| Choquette et al., 2011[15] | Canada | 40 healthy overweight or obese, postmenopausal women 50 to 70 yrs of age assigned to either an exercise+placebo (n=18) or placebo (n=22) group  sedentary  No participation in a systematic/supervised exercise program in the last 5 years | 3 days/wk, 1 h/session: 30 min. aerobic exercise (70-85% HRR on cycle or treadmill) plus 30 min resistance training (4 sets, 4-6 reps, 60-85% 1RM), for 6 months | DEXA (GE Lunar Prodigy) at the LS & FN |
| Englund et al., 2005[16] | Sweden | 40 postmenopausal women 66 to 87 yrs of age assigned to either an exercise (n=21) or control (n=19) group  Subjects inactive | 2 days/wk, 50 min/session: aerobic walk, jog or stepping (10 min), strengthening (2 sets, 8-12 reps for each muscle group, 12 min), dynamic & static balance & coordination exercises (5 min) plus warm-up & cool down, for 47 wks | DEXA (Lunar DPX-L) at the LS & FN |
| Going et al., 2003[17] | United States | 266 healthy, postmenopausal women 40 to 65 yrs of age assigned to an exercise+HT (n=71), exercise+no HT (n=71), control+HT (n=65), or control+no HT (n=59) group  Less than 120 minutes of physical activity per week; no weightlifting or similar activity | 3 days/wk, Weightlifting (7 exercises, 2 sets, 6-8 reps, 70-80% 1RM), Aerobic weight-bearing activity circuit (20-25 min of walk, jog, skip, hop, & stair climb/step box with weighted vest) at 60% of MHR, for 1 yr | DEXA (Lunar DPX-L) at the LS & FN |
| Grove & Londeree, 1992[18] | United States | 15 healthy, sedentary, early postmenopausal women 49 to 64 yrs of age assigned to a low impact exercise (n=5), high impact exercise (n=5) or control (n=5) group  Not active during the last year | 3 days/wk, 1 h/session (15-20 min warm-up, 20 min of either low impact or high impact activities, 15 min cool down), for 1 yr | DPA (Lunar DP3) at the LS |
| Hong, 2004[19] | China | 110 healthy women 65 to 74 yrs of age assigned to a Tai Chi (n=28), resistance training ( n=29) or control (n=30) group  Not exercising regularly | 3 days/wk: Tai Chi: Yang style, 24 forms, 45 min; Resistance Training: 1 set, 30 reps, 7 exercises, therabands used for resistance; for 12 months | DEXA (Hologic QDR 4500 Elite) at the LS & FN |
| Iwamoto et al., 2001[20] | Japan | 28 postmenopausal women 53 to 77 yrs of age assigned to either an exercise (n=8) or control (n=20) group  No engagement in physical activity in the previous 5 years | Participants were provided with a pedometer & encouraged to increase their daily step count by 30% plus 2 days/wk of gymnastic (resistance) training, 2 sets, 15 reps, 4 exercises, for 2 yrs | DEXA (Norland XR-2) at the LS |
| Jessup et al., 2003[20] | United States | 18 women, 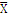 ± SD, 69.2 + 3.5 yrs of age assigned to either an exercise (n=9) or control (n=9) group  Not participating in regular exercise for the previous 12 months | 3 days/wk, 60-90 min/session, Strength Training (8-10 reps at 50-75% 1RM) and 30-45 min walking, stair climbing, & balance exercises wearing weighted vests, for 32 weeks | DEXA (Norland Excell) at the LS & FN |
| Kemmler et al., 2010[22] | Germany | 227 women assigned to either an exercise (n=115, 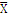 ± SD= 68.9 + 3.9 yrs of age) or placebo (n=112, 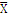 ± SD= 69.2 + 4.1 yrs of age) group  No participation in exercise in the past 2 years or athletic history in the last decade | 2, 60-min supervised classes (20 min aerobic dance at 70-85% max VO_2_; 5 min balance training; functional gymnastics/isometric training/stretching: 1-3 sets, 10-15 reps, 6 exercises; 2-3 sets of 3 upper body exercises (10-15 reps, 9 exercises) and 2 40-min home sessions (1-2 sets, 10-15 reps, 8-11 exercises), for 18 months | DEXA (Hologic QDR 4500 Discovery upgrade) at the LS & FN |
| Kerr et al., 1996[23] | Australia | 42 postmenopausal women assigned to either an endurance strength (n=19, 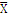 ± SD= 55.7 + 4.7 yrs of age) or a strength (n=23, 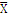 ± SD=58.4 + 3.7 yrs of age) group (one side of body was exercised; the other side was control)  Not exercising more than 3 h/wk at a high intensity; no racquet sports or weight training in last 5 years | 3 days/wk, 3 sets,12 exercises; Endurance: 45-60 min/session, 8 reps, 60% 1RM for leg, 40% 1RM for arm; Strength: 20-30 min/session, 20 reps, 20% 1RM for leg, 10% 1RM for arm; for 1 yr | DEXA (Hologic QDR 2000) at the FN |
| Kerr et al., 2001[24] | Australia | 90 postmenopausal women 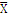 ± SD= 60 + 5 yrs of age assigned to a strength (n=24), fitness (n=30) or control (n=36) group  Not exercising for more than 2 hr/wk at a moderate intensity; no weight training in last 5 years | 3 days/wk, 1 h/session, 3 sets, 9 exercises; Strength: 8 reps, Fitness: 40 seconds per station plus 40 seconds stationary cycling, for 2 yrs | DEXA (Hologic QDR 4500) at the LS & FN |
| Liu-Ambrose et al., 2004[25] | Canada | 98 women 75 to 85 yrs of age assigned to a resistance training (n=32), agility training (n=34) or stretching (control) (n=32) group  Not exercising regularly more than 2 days/wk | 2 days/wk, 50-min/session, Resistance: 2 sets of 10-15 reps at 50-85% 1RM, 9 exercises; Agility: improve balance, coordination & reaction time via ball games, relays, dance & obstacle courses, for 25 wks | DEXA (Hologic 4500) at the FN |
| Marques et al., 2011[27] | Portugal | 71 women 61 to 83 yrs of age assigned to a resistance (n=23), aerobic (n=24 or control (n=24) group  Not engaged in regular exercise training in the last year | 3 days/wk, 60 min/session, Aerobic: 50-85% of HRR for 35-40 min of each session: step, skip, walk, jog, dance, aerobic, step; Resistance: 2 sets, 6-15 reps, 50-85% 1RM, 8 exercises, for 32 wks | DEXA (Hologic QDR 4500A) at the FN |
| Marques et al., 2011[26] | Portugal | 49 women assigned to either an exercise (n=27, 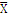 ± SD=70.1 + 5.4 yrs of age) or control (n=22, 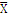 ± SD=68.2 + 5.7 yrs of age) group  Not engaged in regular exercise training in the last year | 2 days/wk, 60 min/session, consisting of 15 min weight-bearing activities, 10 min muscular endurance (3 sets, 8-15 reps), balance & agility training, for 32 wks | DEXA (Hologic QDR 4500A) at the LS & FN |
| Martin & Notelovitz, 1993[28] | United States | 55 postmenopausal women assigned to a 30 min exercise (n=20,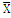 ± SD=60.3 + 7.8 yrs of age), 45 min exercise (n=16, 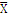 ± SD=57.8 + 7.1 yrs of age) or control (n=19, 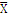 ± SD=56.7 + 6.9 yrs of age) group  Not involved in any aerobic or strength training program in the last 12 months | Aerobic exercise, 3 days/wk, 30 or 45 min/session, 70-85% MHR on treadmills, for 12 months | DPA (Lunar DP3) at the LS |
| Nelson et al., 1994[29] | United States | 39 sedentary, postmenopausal women 50 to 70 yrs of age assigned to either an exercise (n=20) or control (n=19) group  Not strength training and less than 20 min of aerobic exercise 2 days/wk | 2 days/wk of strength training, 45 min/session, 3 sets, 8 reps, 80% 1RM, 5 exercises, for 1 yr | DEXA (Lunar DPX) at the LS & FN |
| Newstead et al., 2004[30] | United States | 49 postmenopausal women 50 to 65 yrs of age assigned to either an exercise (n=25) or control (n=28) group  No current involvement in regular aerobic exercise and/or weight training exercise | 3 jumping sessions/wk on the floor and from aerobic steps (4 and 6 inches), 25-200 jumps/session, for 1 yr | DEXA (Hologic QDR 1500) at the LS & FN |
| Prince et al., 1995[31] | Australia | 66 postmenopausal women 50 to 70 yrs of age assigned to either a calcium+exercise (n=31) or calcium only (n=35) group  Not exercising more than 1 h/wk in last year | 4 h/wk of weight-bearing exercise (2 h classes, 2 h walking on own) at 60% of MHR, for 2 yrs | DEXA (Hologic QDR 1000) at the LS & FN |
| Rhodes et al., 2000[32] | Canada | 38 healthy, sedentary women 65 to 75 yrs of age assigned to either an exercise (n=20) or control (n=18) group  No regular exercise of more than 30 min 3 day/wk; not actively engaged in an organized activity program | Resistance training, 3 days/wk, 1 h/session, 3 sets, 8 reps, 75% 1RM, 6 exercises, for 1 yr | DEXA (Lunar DPX) at the LS & FN |
| Wu et al., 2006[33] | Japan | 64 healthy, postmenopausal women 45 to 60 yrs of age assigned to either a placebo+walking (n=31) or placebo only (n=33) group  Sedentary | 3 days/wk, 45-min/session, walking at a speed of 5-6 km/hr, monitored by a pedometer, for 24 wks | DEXA (Hologic QDR-4500A) at the LS & FN |

Notes: BMD, bone mineral density; DEXA, dual-energy x-ray absorptiometry; DPA, dual photon absorptiometry; FN, femoral neck; LS, lumbar spine; yrs, years; min, minute(s); h, hour(s); wks, weeks; wk, week; km, kilometer; RM, repetition maximum; reps, repetitions; VO_2max_, maximum oxygen consumption; MHR, maximum heart rate; HRR, heart rate reserve; HT, hormone therapy; Description of groups limited to those that met the inclusion criteria for the current meta-analysis; Description of BMD assessment limited to the primary outcomes of the current meta-analysis (FN and LS). Number of subjects limited to those in which final BMD assessments were available.
