# Supplementary material for: Effects of ground and joint reaction force exercise on lumbar spine and femoral neck bone mineral density in postmenopausal women: a meta-analysis of randomized controlled trials
Source: BMC Musculoskelet Disord. 2012 Sep 20;13:177. doi: 10.1186/1471-2474-13-177 (PMC3489866; doi:10.1186/1471-2474-13-177)
Supplement: Additional file 3 — Table of moderator analyses results for FN and LS BMD. This additional file provides a table of results for all moderator analyses that were conducted for categorical variables and changes in femoral neck and lumbar spine bone mineral density. [file 1471-2474-13-177-S3.doc]

Additional File 3. Table of moderator analyses results for FN and LS BMD.

|  | FN | | | LS | | |
| --- | --- | --- | --- | --- | --- | --- |
| Variable | ES (#) | 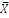 + SE | Q_b_(*p*) | ES (#) | 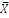 + SE | Q_b_(*p*) |
| Country  - USA  - Other | 5  23 | 0.491 + 0.208  0.242 + 0.109 | 1.11 (0.29) | 9  19 | 0.308 + 0.144  0.112 + 0.120 | 1.10 (0.30) |
| Type of Control  - Nonintervention  - Other | 13  15 | 0.363 + 0.187  0.255 + 0.095 | 0.27 (0.60) | 15  13 | 0.279 + 0.155  0.102 + 0.111 | 0.87 (0.35) |
| Matching  - Yes  - No | 8  20 | 0.206 + 0.120  0.373 + 0.132 | 0.88 (0.35) | 18  10 | 0.141 + 0.147  0.212 + 0.124 | 0.13 (0.72) |
| Sequence generation  - Yes  - No  - Unclear | 28  --  -- | 0.288 + 0.095  --  -- | -- | 28  --  -- | 0.179 + 0.093  --  -- | -- |
| Allocation concealment  - Yes  - No  - Unclear | --  --  27 | --  0.290 + 0.099 | -- | --  --  28 | --  --  0.179 + 0.093 | -- |
| Blinding  - Yes  - No  - Unclear | 27  --  -- | 0.308 + 0.098  --  -- | -- | 27  --  -- | 0.190 + 0.098  --  -- | -- |
| Incomplete outcome data  - Yes  - No  - Unclear | 13  --  15 | 0.274 + 0.119  --  0.314 + 0.153 | 0.04 (0.84) | 13  --  15 | 0.281 + 0.111  --  0.080 + 0.144 | 1.22 (0.27) |
| Outcome reporting bias  - Yes  - No  - Unclear | --  --  25 | --  --  0.285 + 0.104 | -- | --  --  25 | --  --  0.096 + 0.466 | -- |
| Type of analysis  - Per protocol  - Intention to treat | 23  5 | 0.305 + 0.110  0.231 + 0.196 | 0.11 (0.74) | 25  3 | 0.156 + 0.103  0.348 + 0.222 | 0.61 (0.44) |
| Sample size estimate  - Yes  - No | 15  13 | 0.167 + 0.124  0.441 + 0.152 | 1.96 (0.16) | 15  13 | 0.095 +0.114  0.308 + 0.152 | 1.27 (0.26) |
| Funding for study  - Yes  - No | 23  5 | 0.240 + 0.072  1.051 + 0.641 | 1.58 (0.21) | 20  8 | 0.100 + 0.078  0.662 + 0.372 | 2.20 (0.14) |
| Adverse events  - Yes  - No | --  14 | --  0.369 + 0.130 | -- | 9  3 | 0.134 + 0.306  0.319 + 0.148 | 0.30 (0.59) |
| HT  - Yes  - No | 20  -- | 0.145 + 0.149  -- | -- | --  18 | --  0.256 + 0.118 | -- |
| Osteoporosis  - Yes  - No | --  7 | --  0.022 + 0.108 | -- | --  5 | --  0.044 + 0.124 | -- |
| Smoking  - Yes  - No | --  7 | --  0.356 + 0.161 | -- | --  9 | --  0.123 + 0.104 | -- |
| Alcohol consumption  - Yes  - No | --  -- | --  -- | -- | --  -- | --  -- | -- |
| Change in exercise  - Increase  - Decrease  - No change | --  --  8 | --  --  0.167 + 0.161 | --  -- | --  --  6 | --  --  0.223 + 0.130 | -- |
| Some Prior Exercise Allowed  - Yes  - No | 14  14 | 0.193 + 0.084  0.428 + 0.186 | 1.33 (0.25) | 9  19 | 0.042 + 0.091  0.278 + 0.140 | 2.02 (0.16) |
| Calcium administered  - Yes  - No | 10  5 | 0.308 + 0.120  0.118 + 0.122 | 1.23 (0.27) | 12  3 | 0.147 + 0.105  0.626 + 0.575 | 0.68 (0.41) |
| Vitamin D administered  - Yes  - No | 4  3 | 0.544 + 0.346  0.071 + 0.146 | 1.58 (0.21) | 7  -- | 0.338 + 0.151  -- | -- |
| Type of exercise  - Aerobic  - Strength  - Aerobic + strength | 4  13  9 | 0.130 + 0.125  0.354 + 0.209  0.356 + 0.139 | 1.75 (0.42) | 7  10  10 | 0.089 + 0.159  0.225 + 0.237  0.211 + 0.119 | 0.43 (0.81) |
| Exercise delivery (aerobic)  - Supervised  - Unsupervised  - Supervised + Unsupervised | --  --  -- | --  --  -- | -- | 5  --  -- | 0.238 + 0.233  --  -- | -- |
| Exercise delivery (strength)  - Supervised  - Unsupervised  - Supervised + Unsupervised | 10  --  3 | 0.393 + 0.258  --  0.290 + 0.429 | 0.04 (0.84) | 7  --  3 | 0.342 + 0.374  --  0.031 + 0.159 | 0.58 (0.45) |
| Reaction forces  - Ground  - Joint  - Both | 8  12  8 | 0.088 + 0.091  0.420 + 0.222  0.398 + 0.152 | 4.12 (0.12) | 9  10  9 | 0.056 + 0.100  0.190 + 0.244  0.253 + 0.125 | 1.6 (0.46) |
| Instrumentation  - Hologic  - Lunar | 15  6 | 0.220 + 0.089  0.260 + 0.150 | 0.05 (0.82) | 11  8 | -0.025 + 0.133  0.126 + 0.121 | 0.70 (0.40) |

Notes: Data reported as standardized effect size (g); FN, femoral neck; LS, lumbar spine; BMD, bone mineral density; ES(#),number of effect sizes; Z(*p*), *z-*score and alpha value;
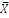
 + SE, mean + standard error; Q_b_(*p*), between-group difference (Q_b_) and alpha value (*p*); --, Insufficient data reported (< 3 ES’s).
