# Supplementary material for: Effects of ground and joint reaction force exercise on lumbar spine and femoral neck bone mineral density in postmenopausal women: a meta-analysis of randomized controlled trials
Source: BMC Musculoskelet Disord. 2012 Sep 20;13:177. doi: 10.1186/1471-2474-13-177 (PMC3489866; doi:10.1186/1471-2474-13-177)
Supplement: Additional file 4 — Table of meta-regression results for changes in FN and LS BMD. This additional file provides a table of results for all regression analyses that were conducted for changes in femoral neck and lumbar spine bone mineral density. [file 1471-2474-13-177-S4.doc]

Additional File 4. Table of meta-regression results for changes in FN and LS BMD.

|  | FN | | | LS | | |
| --- | --- | --- | --- | --- | --- | --- |
| Variable | ES (#) | 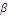_1_ + SE | *Z(p*) | ES (#) | 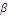_1_ + SE | Z(*p*) |
| Year of publication | 28 | 0.006 + 0.018 | 0.33(0.74) | 28 | -0.011 + 0.018 | -0.63(0.53) |
| Dropouts (%) | 24 | -0.0006 + 0.010 | -0.06(0.95) | 23 | -0.010 + 0.008 | -1.12(0.26) |
| Age (years) | 26 | 0.017 + 0.013 | 1.35(0.18) | 26 | 0.033 + 0.014 | **2.28(0.02)*** |
| Postmenopausal (years) | 21 | 0.010 + 0.010 | 0.94(0.35) | 19 | 0.033 + 0.013 | **2.54(0.01)*** |
| Aerobic exercise  - Length (weeks)  - Frequency (days/week)  - Intensity (% VO_2max_ )  - Duration (min/session)  - Minutes of training/week  - Compliance (%) | 4  3  --  --  --  -- | 0.003 + 0.004  0.04 + 0.20  --  --  --  -- | 0.78(0.43) 0.19(0.85)  --  --  --  -- | 7  7  3  6  6  5 | 0.012 + 0.015  0.047 + 0.301  -0.002 + 0.024  -0.038 + 0.017  -0.013 + 0.006  -0.049 + 0.133 | 0.81(0.42)  0.15(0.88)  -0.08(0.93)  **-2.30(0.02)***  **-2.30(0.02)***  -0.37(0.72) |
| Strength exercise  - Length (weeks)  - Frequency (days/week)  - Intensity of training  - Sets (#)  - Repetitions (#)  - Rest between sets (sec.)  - Exercises (#)  - Compliance (%) | 13  13  6  11  8  4  13  9 | -0.002 + 0.01  -0.154 + 0.170  0.03 + 0.02  -0.080 + 0.237  -0.027 + 0.087  -0.004 + 0.-023  0.060 + 0.060  -0.098 + 0.056 | -0,.20(0.84)  -0.91(0.36)  **1.63(0.10)***  -0.34(0.73)  -0.32(0.75)  -0.16(0.87)  1.00(0.32)  **1.75(0.08)**** | 10  10  4  8  7  3  10  6 | -0.010 + 0.011  -0.153 + 0.186  -0.206 + 0.071  -0.120 + 0.211  -0.003 + 0.041  0.005 + 0.04  0.047 + 0.066  0.148 + 0.049 | -0.92(0.36)  -0.82(0.41)  **2.86(0.004)***  -0.57(0.57)  -0.08(0.94)  0.13(0.90)  0.71(0.47)  **3.03(0.002)*** |
| Aerobic + strength exercise  - Length (weeks)  - Frequency (days/week)  - Compliance (%) | 9  8  7 | -0.006 + 0.006  0.172 + 0.117  -0.021 + 0.006 | -0.94(0.35)  1.47(0.14)  **-3.37(0.0008)*** | 10  9  7 | 0.002 + 0.005  0.205 + 0.164  -0.016 + 0.006 | 0.56(0.57)  1.25(0.21)  **-2.67(0.008)*** |
| Load rating | 21 | -0.0008 + 0.001 | -0.77(0.44) | 24 | -0.0001 + 0.0009 | -0.13(0.90) |
| Baseline BMD | 27 | -0.313 + 1.06 | -0.30(0.77) | 28 | -0.312 + 0.637 | -0.49(0.62) |
| Aerobic fitness (Δ) | 4 | -0.327 + 0.213 | -1.53(0.13) | 8 | -0.315 + 0.180 | **-1.75(0.08)**** |
| Dynamic balance (Δ) | 5 | -0.196 + 0.315 | -0.62(0.53) | 3 | 0.591 + 0.382 | 1.55(0.12) |
| Static balance (Δ) | 5 | 0.788 + 0.422 | **1.87(0.06)**** | 5 | 0.385 + 0.177 | **2.17(0.03)*** |
| Calcium intake (Δ) | 5 | 0.423 + 2.39 | 0.18(0.86) | 7 | -2.179 + 0.271 | -0.80(0.42) |
| Vitamin D intake (Δ) | -- | -- | -- | -- | -- | -- |
| Strength – lower (Δ) | 11 | -0.058 + 0.205 | -0.28(0.78) | 10 | -0.010 + 0.161 | -0.62(0.54) |
| Strength – upper (Δ) | 7 | -0.474 + 0.392 | -1.21(0.23) | 9 | -0.089 + 0.192 | -0.46(0.64) |
| BMI (Δ) | 11 | -0.970 + 0.212 | **-4.57(<0.0001)*** | 13 | -0.637 + 0.141 | **-4.51(<0.0001)*** |
| Body weight (Δ) | 14 | -0.417 + 0.125 | **-3.32(0.0009)*** | 18 | -0.190 + 0.087 | **-2.18(0.03)*** |
| LBM (Δ) | 8 | -0.025 +0.089 | -0.28(0.78) | 8 | 0.400 + 0.241 | **1.66(0.10)**** |
| Percent fat (Δ) | 5 | -0.984 +0.361 | **-2.72(0.006)*** | 5 | -0.782 + 0.274 | **-2.85(0.004)*** |
| Fat mass (Δ) | 6 | 0.107 + 0.172 | -0.62(0.53) | 4 | 0.262 + 0.267 | 0.99(0.32) |

Notes: Notes: ;FN, femoral neck; LS, lumbar spine; BMD, bone mineral density; VO_2max_ , maximum oxygen consumption*;* BMI, body mass index; LBM, Lean body mass; min, minutes; Δ, changes in predictor variable; ES, effect size; #, number;
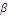
1 + SE, slope + standard error; *Z(p*), *z-*score and alpha value; *, statistically significant (*p* < 0.05) --, **, trend for statistical significance (>0.05 to < 0.10); Insufficient data reported (< 3 ES’s).
